# Supplementary figures and images for: A new specimen of Ziphiidae (Cetacea, Odontoceti) from the late Miocene of Denmark with morphological evidence for suction feeding behaviour
Source: R Soc Open Sci. 2019 Oct 30;6(10):191347. doi: 10.1098/rsos.191347 (PMC6837206; doi:10.1098/rsos.191347)

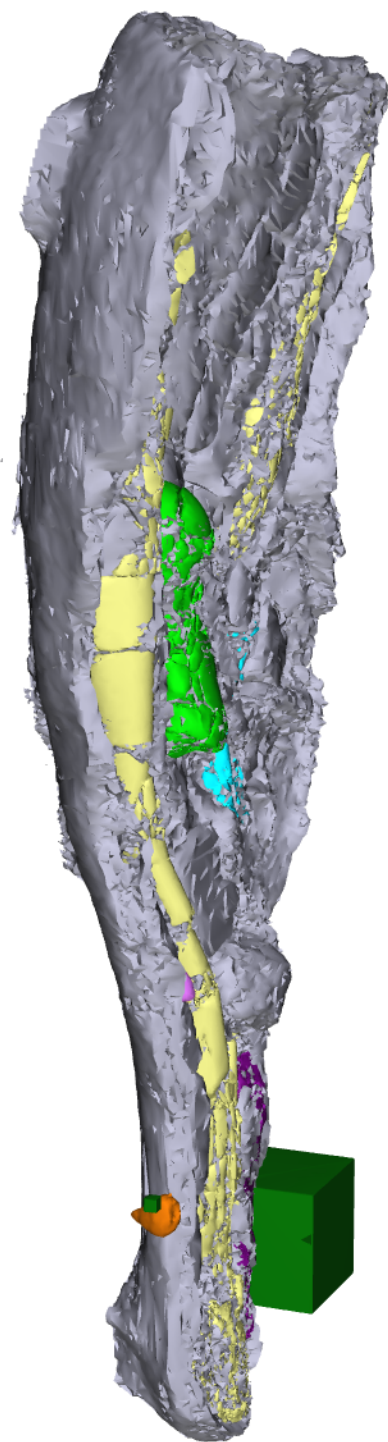

Supplement: Dataset [file rsos191347supp1.pdf]
